# Mitochondria-Targeted Mesoporous Organic Silica Nanoplatforms for Overcoming Cisplatin Resistance by Disturbing Mitochondrial Redox Homeostasis

**DOI:** 10.3389/fchem.2022.875818

**Published:** 2022-05-09

**Authors:** Bin Lv, Jingru Ma, Yumeng Wang, Xinyu Qu, Junjun Qiu, Keqin Hua

**Affiliations:** ^1^ Department of Gynecology, Obstetrics and Gynecology Hospital, Fudan University, Shanghai, China; ^2^ Shanghai Key Laboratory of Female Reproductive Endocrine Related Diseases, Shanghai, China; ^3^ ENT Institute and Department of Otorhinolaryngology, Eye and ENT Hospital, Fudan University, Shanghai, China

**Keywords:** cisplatin, resistance, mitochondria, redox homeostasis, GSH depletion

## Abstract

Cisplatin (also known as DDP) resistance is one of the biggest challenges in the treatment of ovarian cancer. Recent studies have found that mitochondrion, as a potential target of DDP, participates in drug-related apoptosis and resistance. Overexpressed glutathione (GSH) in resistant cells is involved in protecting mitochondria from DDP or DDP-induced ROS. In this work, triphenylphosphonium (TPP) modified disulfide bond-rich (S-S) mesoporous organic silica nanoplatforms (DMON) were developed to deliver DDP (TPP-DMON@DDP) to mitochondria for overcoming DDP resistance. TPP supported the migration of nanoplatforms to the mitochondria, with consequent depletion of mitochondrial GSH by the S-S bond of DMON, leading to mitochondria in redox dyshomeostasis. These treated cells seemed more susceptible to the DDP released from the nanoplatforms. Significantly increased ROS production, mitochondrial damage, and apoptosis were observed in TPP-DMON@DDP-treated cells. Overall, interference of mitochondrial redox homeostasis provides a new opportunity for improving DDP cytotoxicity against resistant cells.

## Introduction

Epithelial ovarian cancer (EOC) is frequently diagnosed at an advanced stage, and standard care consists of debulking surgery and platinum-based chemotherapy (Kurnit et al., 2021). However, more than half of patients with advanced EOC experience relapse within 5 years and develop drug resistance (Wang et al., 2019). Cis-diamine-dichloroplatinum (II) (known as cisplatin or DDP), the most common platinum drug, is generally considered to primarily provoke nuclear DNA damage by forming DDP-DNA adducts (Dasari and Tchounwou, 2014). Interestingly, some studies have found that only 1% of DDP interacts with nuclear DNA, and DDP exerts prominent cytotoxicity in enucleated cells (Mandic et al., 2003; Yang et al., 2006; Galluzzi et al., 2014). It is indicated that the cytotoxicity of DDP involves cytoplasmic signaling pathways. Accumulating studies suggest that damage involving mitochondria may contribute significantly to the cytotoxicity of DDP (Galluzzi et al., 2014; He et al., 2020; Gong et al., 2021). Activated DDP is a potent electrophile that can react with a nucleophile in the mitochondria, including the mitochondria DNA (mtDNA) and protein sulfhydryl groups, resulting in an increase in reactive oxygen species (ROS) level, reduction of mitochondrial membrane potential, and activation of apoptosis (Cocetta et al., 2019). These findings suggest that the mitochondria are pivotal in the DDP-induced apoptosis, and targeting the mitochondria to deliver DDP may enhance the anti-cancer efficacy.

Recent studies report that the mitochondria participate in DDP resistance in various cancer types (Patel et al., 2019). With the metabolic reprogramming of DDP-resistant cells, the morphology or function of the mitochondria changes (Montopoli et al., 2011). DDP-resistant cells prefer performing oxidative phosphorylation in mitochondria, whereas DDP-sensitive cells preferentially rely on glycolysis (Zampieri et al., 2020). Interestingly, the mitochondria of DDP-resistant cells become more active but less abundant (Kleih et al., 2019) and are better preserved following treatment with DDP than that of DDP-sensitive cells (Zampieri et al., 2020). Treatment with H_2_O_2_ or DDP causes severe mtDNA damage in sensitive cells, but only slight injury to mtDNA is detected in resistant cells (Hirama et al., 2006). Mitochondria in resistant cells have similar tolerance to DDP and ROS, indicating that protective factors against ROS may also prevent the toxicity of DDP. Obviously, glutathione (GSH) overexpressed in resistant cells can not only maintain redox homeostasis by reducing ROS but also inactivate DDP *via* forming adducts (GS-Pt) (Liang et al., 2021). Undoubtedly, overexpressed GSH protects mitochondria from ROS or DDP. Recent studies have reported that increasing mitochondrial ROS can effectively sensitize ovarian cancer cells to DDP (Kleih et al., 2019). Given the high reactivity between ROS and GSH, it is reasonable to assume that GSH in/around mitochondria (mitochondrial GSH) may contribute dramatically to DDP resistance. Furthermore, it can be speculated that the mitochondria in redox dyshomeostasis may be more vulnerable to DDP. Therefore, the delivery of DDP to the mitochondria while consuming GSH may provide a promising pathway for overcoming DDP resistance.

**FIGURE 1:**
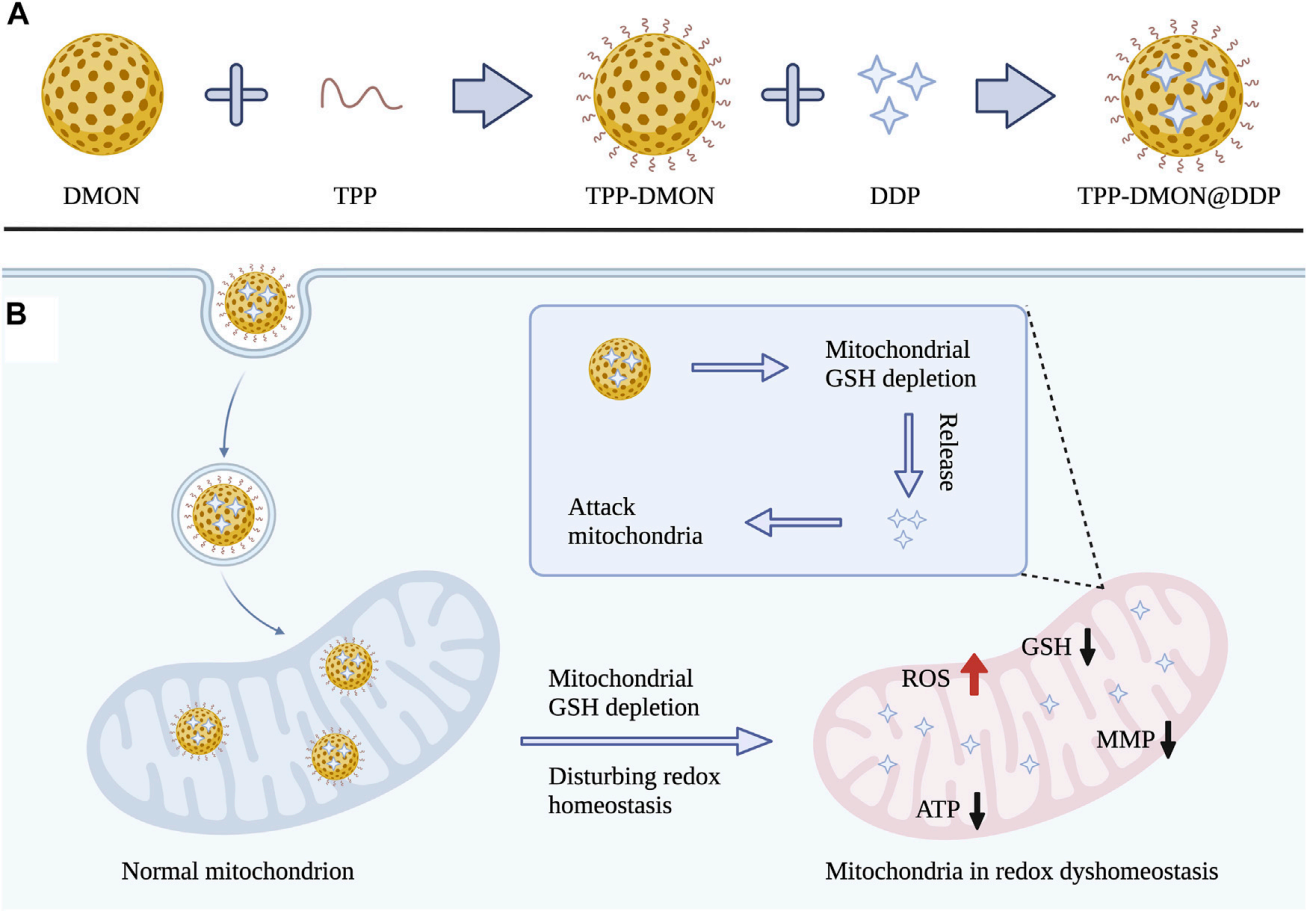
**(A)** Schematic illustration for the synthesis process of TPP-DMON@DDP. **(B)** The mechanism of overcoming DDP resistance by disturbing mitochondrial redox homeostasis and attacking mitochondria.

Herein, we designed disulfide bond-rich (S-S) mesoporous organic silica nanoplatform (DMON) modified with triphenylphosphonium (TPP) as delivery vehicles for loading DDP (TPP-DMON@DDP). As shown in Figure 1B, TPP was decorated on the surface of the DMON, allowing effective mitochondrial localization (Wang et al., 2021). DMON depleted mitochondrial GSH and decomposed themselves, resulting in redox dyshomeostasis of the mitochondria and release of DDP. The mitochondria in redox dyshomeostasis were more vulnerable to DDP, exhibiting robust increases in ROS and decreases in ATP and mitochondrial membrane potential (MMP). Ultimately, this strategy demonstrated promising therapeutic effects on DDP-resistant ovarian cancer cells. In short, the prepared TPP-DMON@DDP directly migrated to the mitochondria and disturbed mitochondrial redox homeostasis, sensitizing the damage of DDP to mitochondria. It provides an innovative strategy for effectively overcoming DDP resistance through well-designed biodegradable nanoplatforms.

## Experimental Section

### Materials

Tetraethoxysilane (TEOS), bis[3-(triethoxysilyl)propyl]tetrasulfide (BTES), N-hydroxysuccinimide (NHS), 1-ethyl-3-(3-dimethylaminopropyl)carbodiimide-HCl (EDC), triethanolamine (TEA), (3-aminopropyl)triethoxysilane (APTES), (3-carboxypropyl) triphenylphosphonium bromide (TPP-COOH), and cisplatin (DDP) were purchased from Sigma-Aldrich. Hexadecyl trimethyl ammonium bromide (CTAB) was purchased from Adamas Reagent. Methyl alcohol, sodium salicylate (NaSal), and sodium chloride (NaCl) were purchased from Sinopharm Chemical Reagent Co., Ltd. Cell Counting Kit-8 (CCK-8) was purchased from TargetMol (Boston, MA, United States). GSH and GSSG Assay Kit, MitoTracker Red CMXRos, Reactive Oxygen Species Assay Kit (2′,7′-dichlorodihydrofluorescein diacetate, DCFH-DA), Mitochondrial Membrane Potential Assay Kit with JC-1, ATP Assay Kit, Calcein-AM and Propidium Iodide (PI) Assay Kit, and Annexin V-FITC Apoptosis Detection Kit were obtained from Beyotime (Shanghai, China). Bax (D2E11) and Bcl-2 (D55G8) Rabbit mAb were provided by Cell Signaling Technology (Beverly, MA, United States). GAPDH was purchased from Abcam (Cambridge, MA, United States). All the chemical reagents were directly employed without purification.

### Synthesis of Dendritic Mesoporous Organic Silica Nanoplatform

The synthesis of DMON is referenced in Liu et al. (2021). In brief, after 0.125 g of TEA was dispersed in 25 ml of deionized water, the solution was transferred to an oil bath (0.5 h, 80°C). Next, the solution was added with 84 mg of NaSal and 380 mg of CTAB. Half an hour later, the above solution was added with 1.6 ml of BTES and 2 ml of TEOS. The solution turned from transparency to yolk yellow gradually. The product was collected by high-speed centrifugation (12,000 rpm, 10 min) and washed with ethanol several times. Afterward, the collected product was re-dispersed in 100 ml of 1% (w/w) NaCl methanol solution with robust stirring for 6 h. Finally, the DMON was obtained by high-speed centrifugation (12,000 rpm, 10 min).

### Synthesis of Dendritic Mesoporous Organic Silica Nanoplatform-NH_2_

First, 20 μl APTES was added into 10 ml DMON ethanol solution and heated to 70°C with reflux for 12 h. Then, the DMON-NH_2_ was collected by centrifugation at 13,000 rpm and washed with ethanol three times. Finally, the DMON-NH_2_ was dispersed in 5 ml PBS solution (pH = 7).

### Synthesis of Triphenylphosphonium-Dendritic Mesoporous Organic Silica Nanoplatform

First, 0.1 g TPP-COOH, 0.3 mmol EDC, and 0.3 mmol NHS were added to 5 ml of the PBS solution (pH = 7), and the mixture solution was stirred for 15 min. Then, 5 ml DMON-NH_2_ solution (pH = 7) was added to the mixture solution and stirred for 12 h. Subsequently, the TPP-DMON was collected by centrifugation at 13000 rpm. Finally, the TPP-DMON was dispersed in a 5 ml PBS solution (pH = 7).

### Synthesis of TPP-DMON@DDP

First, 100 mg of TPP-DMON and 10 mg of DDP were added to a 10 ml solution of saline, and the mixed solution was stirred for 24 h under dark conditions. Then, the TPP-DMON@DDP was separated by centrifuge and washed three times with saline. Finally, the samples were placed in a vacuum oven at 30°C for 4 days to remove any residual solvent. The amount of DDP was measured with ICP-OES (Agilent Technologies, United States).

### The Glutathione Depletion Efficiency of Dendritic Mesoporous Organic Silica Nanoplatform

The DMON was dispersed in GSH solution (10 mM, 10 ml). Then, the mixed solution (1 ml) was collected at different time points (0, 4, 8, 12, 24, and 48 h) and centrifuged (10 min, 13,000 rpm) to acquire the supernatant. A GSH and GSSG detection assay kit was performed to detect GSH concentrations in the supernatants.

### Detection of Cisplatin Release

TPP-DMON@DDP was transferred into a dialysis bag and dispersed into GSH solutions (0, 5, and 10 mM), followed by magnetic stirring at room temperature. The solution was collected at different time points, and the Pt element concentration in the solution was determined by ICP-OES to calculate the release rate of DDP.

### Cell Culture

Human ovarian cancer A2780 cells and acquired DDP-resistant A2780 (A2780/DDP) cells were cultured in Roswell Park Memorial Institute medium (RPMI) 1640 containing 10% fetal bovine serum (FBS) and 1% Penicillin-Streptomycin. All cells were cultured in a humidified atmosphere of 5% CO_2_ and 37°C.

### Cytotoxicity Assays

The cell viability was measured using CCK-8. A2780 cells or A2780/DDP cells were seeds in 96-well plates (2 × 10^3^ cells well^−1^). After cell adherence, the culture media were substituted by fresh media containing DDP at concentrations of 0, 0.1, 1, 2, 4, 8, 16, 32, 64, and 128 ppm for incubation of 48 h. Then, after removing the medium, the cells were washed with PBS and treated with 100 μl of new mix medium containing 10 μl of CCK-8 reagent for 1 h. Finally, cell viability was measured by the absorbance at 450 nm using a microplate spectrophotometer (Bio-TekEL x800, United States).

### Intracellular Glutathione Measurement

Cells were seeds in six-well plates (5 × 10^4^ cells well^−1^) and cultured overnight. Subsequently, cells were incubated with PBS, DDP (2 ppm), DMON@DDP (DDP, 2 ppm), and TPP-DMON@DDP (DDP, 2 ppm). About 48 h later, the treated cells were harvested to investigate GSSG and GSH levels using the GSH and GSSG Assay Kit following standard protocols.

### Cell Uptake and Mitochondrial Localization Assay

A2780/DDP cells were seeded in confocal dishes (5 × 10^3^ cells well^−1^). After incubation overnight, the culture medium was replaced with a fresh medium containing FITC-labeled TPP-DMON@DDP. About 4 h later, cells were treated with MitoTracker Red CMXRos (the mitochondrial fluorescence probe) and Hoechst for 30 min and then imaged by confocal laser scanning microscopy (CLSM) (A1R MP, Nikon, Japan).

### Mitochondrial Membrane Potential Measurement

A2780/DDP cells were inoculated in confocal dishes (5 × 10^3^ cells well^−1^) overnight and then treated with PBS, DDP (2 ppm), DMON@DDP (DDP, 2 ppm), and TPP-DMON@DDP (DDP, 2 ppm) for 48 h. Subsequently, these treated cells were washed with PBS and incubated with JC-1 working solution and Hoechst for 20 min. The data were recorded by CLSM.

### Intracellular ATP Content Measurement

After being inoculated in six-well plates (5 × 10^4^ cells well^−1^) and cultured overnight, A2780/DDP cells were treated with PBS, DDP (2 ppm), DMON@DDP (DDP, 2 ppm), and TPP-DMON@DDP (DDP, 2 ppm) for 48 h. Then, these treated cells were collected and lysed to detect the ATP level using the ATP Assay Kit.

### Intracellular Reactive Oxygen Species Detection

A2780/DDP cells were inoculated in confocal dishes (5 × 10^3^ cells well^−1^) overnight and then treated with PBS, DDP (2 ppm), DMON@DDP (DDP, 2 ppm), and TPP-DMON@DDP (DDP, 2 ppm) for 24 h. Then, these treated cells were washed with PBS and incubated with PBS containing DCFH-DA for 30 min. Finally, the fluorescence intensity was tested by CLSM.

### Calcein-AM/Propidium Iodide and Annexin V-FITC/Propidium Iodide Assay

A2780 cells were inoculated in six-well plates (5 × 10^4^ cells well^−1^) and cultured overnight. Then, the medium was replaced with a fresh medium containing PBS, DDP (2 ppm), DMON@DDP (DDP, 2 ppm), and TPP-DMON@DDP (DDP, 2 ppm). After incubation for 48 h, these cells were harvested and stained with Calcein-AM/PI or Annexin V-FITC/PI fluorescence dye and recorded by CLSM or flow cytometry (CytoFLEX, Beckman, United States).

### Western Blotting Analysis

A2780 cells were inoculated in six-well plates (5 × 10^4^ cells well^−1^) and treated the same as the treatment of the Calcein-AM/PI assay. About 48 h later, these treated cells were harvested and lysed to obtain cellular protein. After protein samples were electrophoresed and transferred to polyvinylidene fluoride (PVDF) membranes (Millipore, United States), these membranes were blocked by 5% BSA for 1 h and then treated with primary antibodies containing Bcl-2 (1:1,000 dilution), Bax (1:1,000 dilution), and GAPDH (1:10,000 dilution). After incubation overnight, the membranes were treated with Goat anti-Rabbit IgG (H+L Secondary antibody (1:5,000 dilution) for 1 h and investigated by an electrochemiluminescence reagent kit following the manufacturer protocols.

### *In Vivo* Toxicity Assay

All procedures of animal experiments were conducted based on the guidelines of the Regional Ethics Committee for Animal Experiments and approved by the Department of Laboratory Animal Science Fudan University (accreditation number: 202011012S).

BALB/c mice (7 weeks, female) were injected intravenously with PBS or TPP-DMON@DDP (DDP, 4 mg kg^−1^) suspended in saline and sacrificed at the predetermined time (3rd or 30th day) to obtain blood for hematological determination.

### *In Vivo* Therapeutic Efficiency

The xenograft tumor model was established by subcutaneously inoculating A2780/DDP cells (5 × 10^6^) into the flank of BALB/c mice (7 weeks, female). When the tumors grew to 100 mm^3^, the tumor-bearing mice were randomly divided into four groups (*n* = 7) and intravenously treated with PBS, DDP, DMON@DDP (DDP, 2 mg kg^−1^), or TPP-DMON@DDP (DDP, 2 mg kg^−1^). After 48 h of the first injection, one mouse was randomly selected from each group, and its tumor was resected for histological analyses. The other mice were given a second intravenous injection with the same drug concentration as the last time. The body weight, tumor length (L), and tumor width (W) of mice were measured every 3 days. Then, tumor volume (V) was calculated with the following equation: $V=L\times W^{2}/2$ .

## Results and Discussion

### Characterization of TPP-DMON@DDP

**FIGURE 2:**
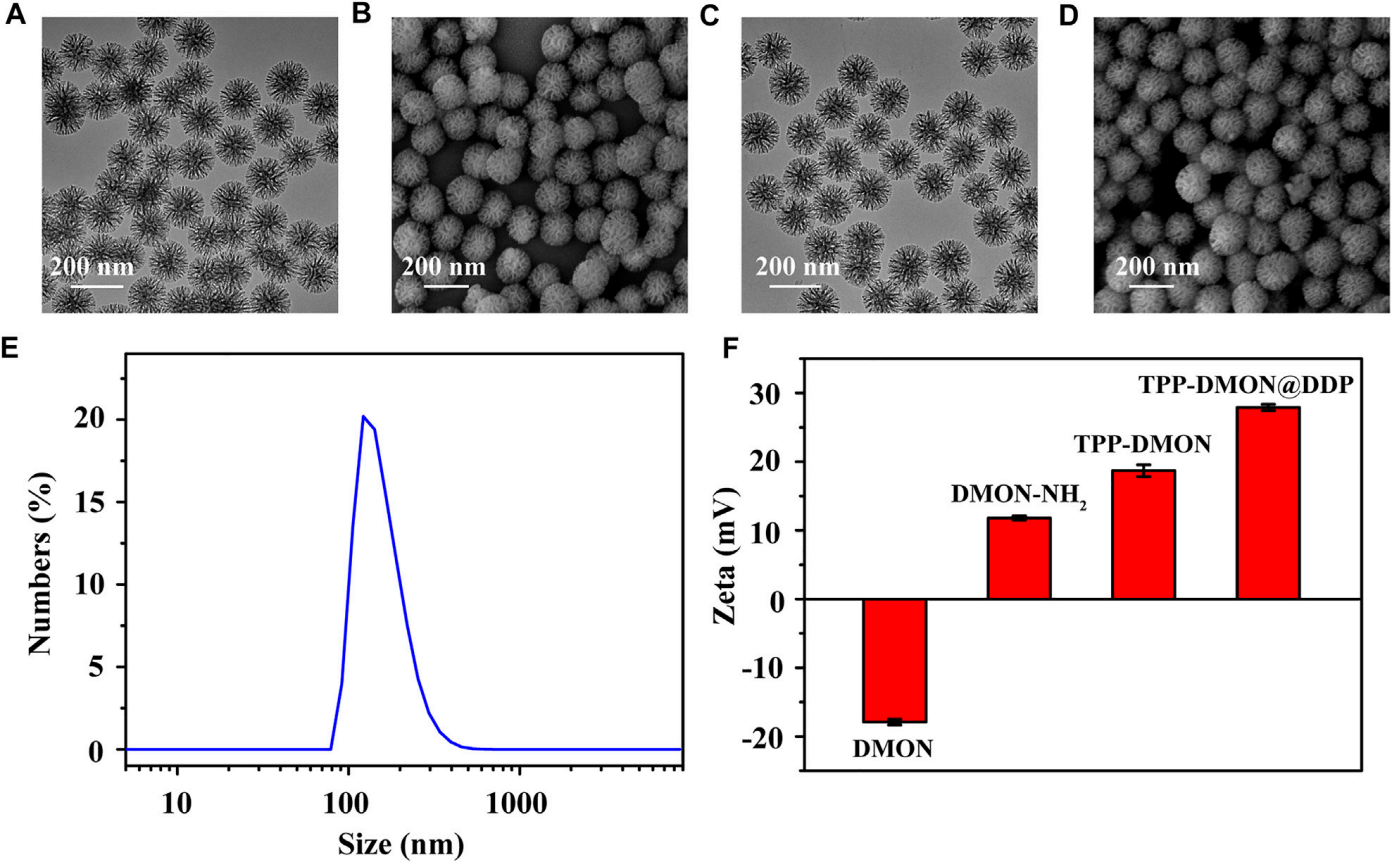
Characteristics of nanoplatforms. TEM **(A)** and SEM **(B)** images of DMON. Scale bar = 200 nm. TEM **(C)** and SEM **(D)** images of TPP-DMON@DDP. **(E)** DLS data of TPP-DMON@DDP in saline. **(F)** Zeta potentials of DMON, DMON-NH_2_, TPP-DMON, and TPP-DMON@DDP.

**FIGURE 3:**
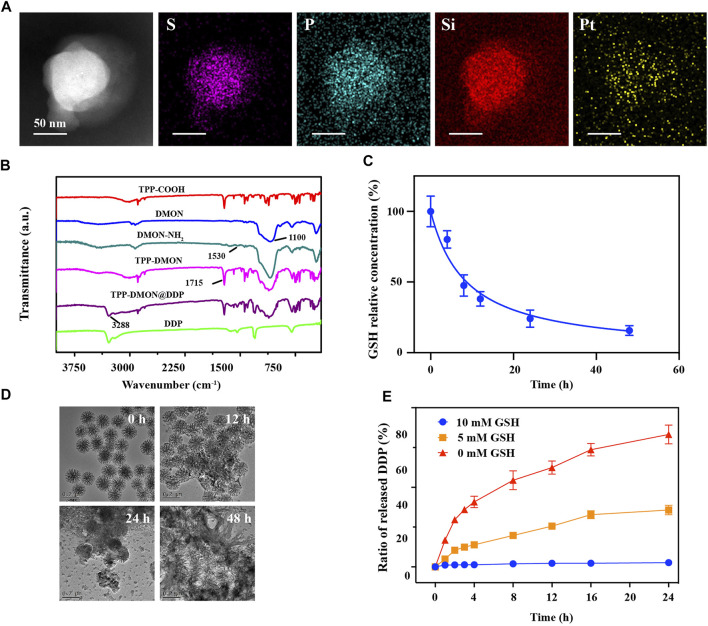
Performance of nanoplatforms in solution. **(A)** Elemental mapping of TPP-DMON@DDP. Scale bar = 50 nm. **(B)** Fourier transform infrared (FT-IR) spectra of DDP, TPP-COOH, DMON, DMON-NH_2_, TPP-DMON, and TPP-DMON@DDP. **(C)** Consumption rate of GSH (10 mM) by DMON. **(D)** TEM images of TPP-DMON@DDP dispersed in GSH solution (10 mM) for 0, 12, 24, and 48 h. **(E)** Release performance of DDP from TPP-DMON@DDP after incubation with different concentrations of GSH.

The TPP-DMON@DDP was prepared as shown in Figure 1A. Clear dendritic pores of DMON were observed in the TEM and SEM images (Figures 2A,B). The surface of DMON was then modified with TPP (TPP-DMON) to obtain the ability to target the mitochondria. Notably, the loading process of DDP in TPP-DMON (TPP-DMON@DDP) was carried out in saline, which provided a feasible chloride concentration to suppress DDP hydrolysis. TEM and SEM images showed that TPP-DMON@DDP maintained a stable structure and good dispersion (Figures 2C,D), which was further confirmed by dynamic light scattering (DLS) and zeta potential measurements (Figures 2E,F). Energy-dispersive X-ray spectroscopy analyses demonstrated representative elements of Si, S, P, and Pt in TPP-DMON@DDP (Figure 3A and Supplementary Figure S1). Fourier transform infrared (FT-IR) spectra of TPP-DMON@DDP displayed absorption signals at 1,100, 1,715, and 3,288 cm^−1^, which were attributed to the characteristic peaks of DMON, TPP, and DDP, respectively (Figure 3B). It demonstrated that TPP was modified on the surface of DMON, and DDP was successfully loaded into DMON. The loading rate of DDP in TPP-DMON@DDP tested by inductively coupled plasma optical emission spectrometry (ICP-OES) was about 4.17 ± 0.23%. According to previous studies, nanoparticles composed of S-S can effectively consume GSH by the redox reaction, leading to self-disintegration (Liu et al., 2021). As expected, the DMON rich in S-S efficiently consumed GSH over time (Figure 3C). The S-S in TPP-DMON@DDP reacted with GSH, causing the collapse of the nanoplatform structure and release of DDP. It depended on the amount of GSH and reaction time (Figures 3D,E).

### Characterization of Cisplatin-Resistant Cells

**FIGURE 4:**
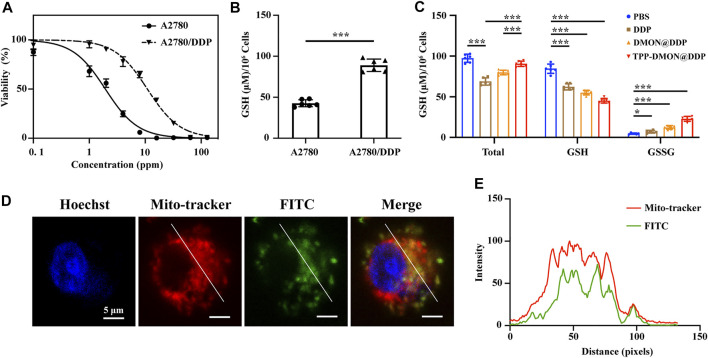
The resistant characteristic of A2780/DDP cells and mitochondria-targeting delivery. **(A)** Cell viability of A2780 and A2780/DDP cells after incubation with DDP. **(B)** Intracellular GSH content in A2780 and A2780/DDP cells (*p < 0.05, **p < 0.01, ***p < 0.001, *n* = 6). **(C)** Intracellular GSH content in A2780/DDP cells after treatment with PBS, DDP, DMON@DDP, and TPP-DMON@DDP (*p < 0.05, **p < 0.01, ***p < 0.001, *n* = 6). **(D)** Distribution of TPP-DMON@DDP in A2780/DDP cells detected by CLSM with Hoechst (blue), MitoTracker (red), and FITC-labeled TPP-DMON@DDP (green). Scale bar = 5 μm. **(E)** Co-localization analysis using ImageJ.

*In vitro* experiments were performed using human ovarian cancer A2780 cells with acquired DDP resistance (A2780/DDP cells). These A2780/DDP cells were derived from A2780 cells that survived under the pressure of persistent exposure to DDP. Prior to all cell experiments, cell viability assays were performed to determine DDP tolerance. As shown in Figure 4A, the therapeutic effect of DDP on A2780/DDP cells was significantly reduced (IC_50_ = 10.91 × 0.67 ppm), whereas that on parental A2780 cells remained sensitive (IC_50_ = 1.94 × 0.17 ppm). According to previous studies, resistant cells generally upregulate intracellular GSH to inactivate DDP with GS-Pt adducts (Levy et al., 2019; Bravo-Cuellar et al., 2020; Xu et al., 2020). As shown in Figure 4B, A2780/DDP cells were characterized by higher expression levels of GSH similar to those previously reported (Xu et al., 2020). Encouraged by the results of the GSH depletion assays in solution, we next investigated the intracellular GSH levels upon the different treatments *in vitro*. Interestingly, both the total-GSH (GSH + GSSG) and GSH levels in A2780/DDP cells decreased after treatment with DDP (Figure 4C). One plausible reason is that GS-Pt adducts cannot be detected by the assay kit (Luo et al., 2020). However, both DMON@DDP- and TPP-DMON@DDP-treated cells showed higher total-GSH levels than DDP-treated cells, with TPP-DMON@DDP-treated being the highest. This suggests that DMON contributed to protecting DDP from GSH inactivation, which could be further improved by mitochondria-targeted delivery. Further, compared to PBS-treated cells, the TPP-DMON@DDP-treated cells had significantly decreased GSH and increased GSSG levels, demonstrating that the decreased GSH might be involved in the reaction with S-S or DDP-induced ROS.

### Mitochondrial Localization

Mitochondria are involved in the regulation of DDP-induced apoptosis (Zhang et al., 2021), and targeted mitochondria delivery can enhance the toxicity of DDP (Gao et al., 2021; Yu et al., 2021). Prior to evaluating the cytotoxicity of TPP-DMON@DDP, we investigated the mitochondrial localization efficiency of nanoplatforms in A2780/DDP cells using confocal laser scanning microscopy (CLSM). As shown in Figures 4D,E, the FITC-labeled (green fluorescence) TPP-DMON@DDP overlapped well with the red fluorescence of MitoTracker Red CMXRos, a commercially mitochondrial probe, albeit with different fluorescence intensities. This indicated that the TPP-DMON@DDP could well localize in the mitochondria.

### Evaluation of Mitochondrial Damage and Reactive Oxygen Species Levels

**FIGURE 5:**
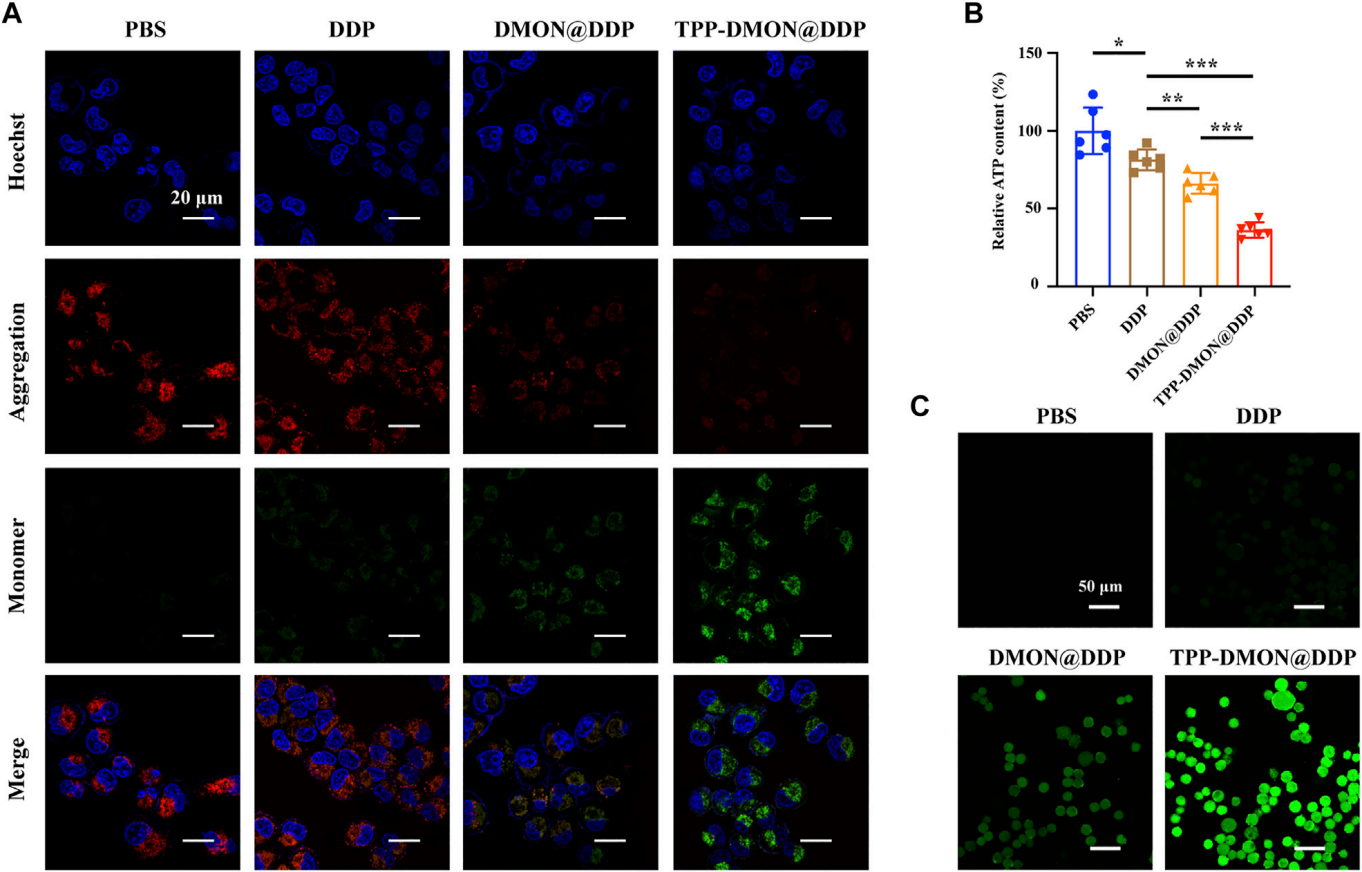
Evaluation of mitochondrial damage and ROS production. **(A)** CLSM images of JC-1 stained A2780/DDP cells after treatment with PBS, DDP, DMON@DDP, and TPP-DMON@DDP. Scale bar = 20 μm. **(B)** Relative intracellular ATP content of A2780/DDP cells after incubation with PBS, DDP, DMON@DDP, and TPP-DMON@DDP (*p < 0.05, **p < 0.01, ***p < 0.001, *n* = 6). **(C)** CLSM images of DCFH-DA stained A2780/DDP cells after exposure to PBS, DDP, DMON@DDP, and TPP-DMON@DDP. Scale bar = 50 μm.

With mitochondrial GSH depletion, DDP is released from nanoplatforms and attacks mitochondria. Mitochondrial damage can be estimated by MMP and intracellular ATP levels (Yang et al., 2020). Then, we measured the MMP using the fluorescent probe JC-1. Red fluorescence from JC-1 aggregates was visible in PBS-treated cells, representing the high membrane potentials (Figure 5A). However, the fluorescence of JC-1 shifted from red to green in cells after treatment with DDP-based treatment, suggesting that the mitochondria were potential targets of DDP. Furthermore, DMON@DDP-treated cells exhibited higher green fluorescence intensity than DDP-treated cells, indicating that depletion of GSH could augment DDP-induced mitochondrial damage. We also found that the mitochondrial damage was further increased when DMON@DDP was modified with TPP to target the mitochondria. In addition, the results of intracellular ATP measurement were similar to those of MMP detection (Figure 5B). Although A2780/DDP cells had a modest decrease in intracellular ATP after treatment with DDP, the decrease was greater after exposure to DMON@DDP or TPP-DMON@DDP. The redox dyshomeostasis caused by the absence of reductive species tends to amplify the damaging effects of ROS (Li Y. et al., 2020), and the serious mitochondrial damage induced by TPP-DMON@DDP might be related to mitochondrial redox dyshomeostasis.

Previous studies have reported that DDP mainly attacks mtDNA and mitochondrial protein sulfhydryl groups, resulting in disturbance of the respiratory chain and elevating ROS generation (Gao et al., 2021). Thus, we detected the intracellular ROS production using DCFH-DA. As shown in Figure 5C, the green fluorescence of DCF was observed in all DDP-based treatments, suggesting the production of ROS. Obviously, DMON@DDP, consuming GSH by DMON, induced increased ROS production, further enhanced by targeted mitochondrial delivery. The additional increase in ROS is generally considered to cause depolarization of MMP (Liu et al., 2021), which might be the cytotoxic mechanism of TPP-DMON@DDP.

### Evaluation of Therapeutic Efficiency

**FIGURE 6:**
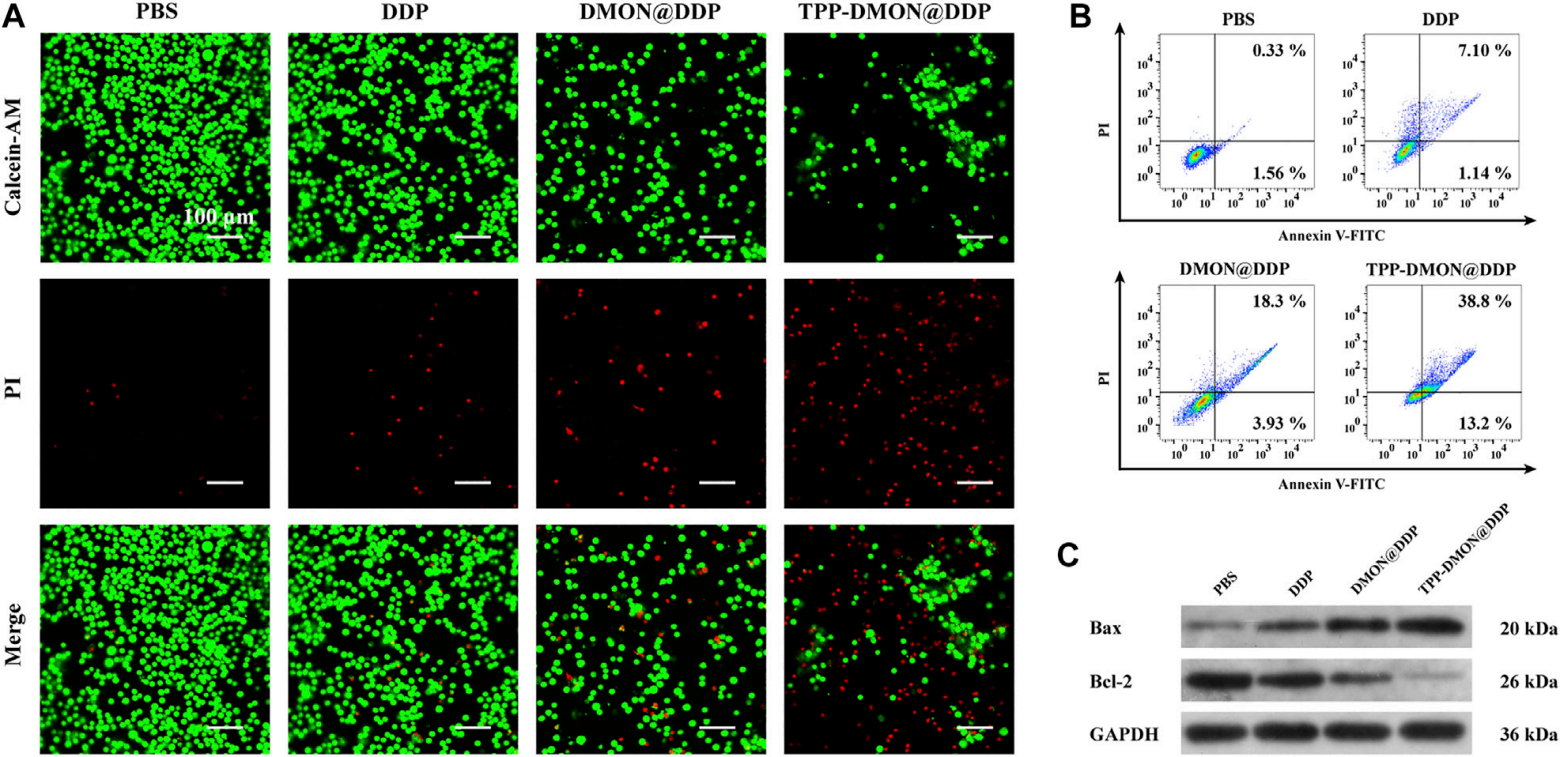
Assessment of the therapeutic efficiency of different treatment. **(A)** CLSM images of Calcein-AM/PI stained A2780/DDP cells after treatment with PBS, DDP, DMON@DDP, and TPP-DMON@DDP. Scale bar = 100 μm. **(B)** Flow cytometry measuring apoptosis of A2780/DDP cells after incubation with PBS, DDP, DMON@DDP, and TPP-DMON@DDP. **(C)** Western blotting analysis of Bax and Bcl-2 expression.

Next, we assessed the therapeutic efficiency of TPP-DMON@DDP against A2780/DDP cells. As shown in Calcein-AM/PI double-stained assays, fewer live cells (green fluorescence) and more dead cells (red fluorescence) were observed in the TPP-DMON@DDP group, followed by the DMON@DDP group (Figure 6A). These results suggested that GSH depletion contributed to increasing the cytotoxicity of DDP against A2780/DDP cells, which was a feasible strategy for reversing DDP resistance. However, we found that the toxicity of DDP was further enhanced by the addition of targeted mitochondria, demonstrating that the consumption of mitochondrial GSH was much more effective in sensitizing DDP. Encouraged by the promising therapeutic effect of TPP-DMON@DDP on A2780/DDP cells, normal cells were employed to further understand the cytotoxicity mechanism. As shown in Supplementary Figure S2, TPP-DMON@DDP exhibited severe cytotoxicity to A2780/DDP cells but milder to normal cells. The possible mechanism was that the high GSH content of A2780/DDP cells facilitated the degradation of TPP-DMON@DDP and the release of DDP to perform cytotoxicity.

It is generally considered that the cytotoxicity of DDP mainly depends on apoptosis (Li C. et al., 2020). Therefore, we conducted the Annexin V-FITC/PI staining assays to evaluate apoptosis using flow cytometry. As shown in Figure 6B, the apoptotic populations exposed to PBS, DDP, DMON@DDP, and TPP-DMON@DDP were 1.89%, 8.24%, 22.23%, and 52%, respectively. This result of apoptosis was consistent with the mitochondrial damage investigation. To understand the mechanism of apoptosis caused by TPP-DMON@DDP, we determined the expression of Bax and Bcl-2, which are involved in executing the mitochondria-mediated apoptosis pathway (Alarifi et al., 2017; Lv et al., 2020). As shown in Figure 6C, the upregulated expression of Bax and downregulated expression of Bcl-2 were detected in DDP-, DMON@DDP-, and TPP-DMON@DDP-treated cells. These results were consistent with the above cell viability assays, demonstrating that TPP-DMON@DDP induced apoptosis in A2780/DDP cells through the mitochondrial pathway.

Encouraged by the promising therapeutic effect of TPP-DMON@DDP on A2780/DDP cells, normal cells were employed to further understand the cytotoxicity mechanism. As shown in Supplementary Figure S2, TPP-DMON@DDP exhibited severe cytotoxicity to A2780/DDP cells but milder to normal cells. The possible mechanism was that the high GSH content of A2780/DDP cells facilitated the degradation of TPP-DMON@DDP and the release of DDP to perform cytotoxicity.

### *In Vivo* Therapeutic Assessment

Encouraged by the promising *in vitro* therapeutic efficiency, we next assessed the *in vivo* feasibility of TPP-DMON@DDP. After intravenous injection of TPP-DMON@DDP in healthy BALB/c mice, the TPP-DMON@DDP-treated mice showed negligible changes in body weights and hematological parameters compared to PBS-treated mice (Supplementary Figures S3, S4).

**FIGURE 7:**
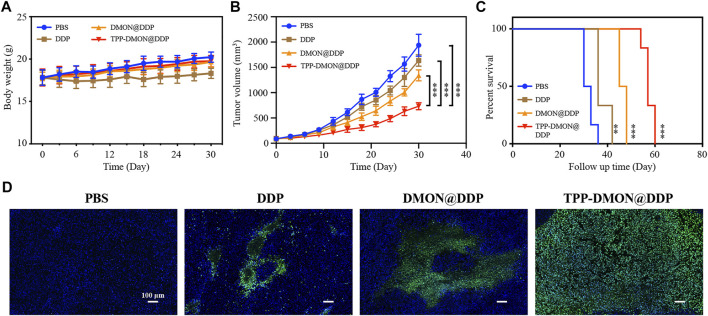
*In vivo* investigation of therapeutic efficiency. **(A)** Mice body weight recorded during the follow-up period. **(B)** Time-dependent relative tumor volume changes after treatment with PBS, DDP, DMON@DDP, and TPP-DMON@DDP. Statistical analysis was conducted with Student’s *t*-tests (*p < 0.05, **p < 0.01, and ***p < 0.001). **(C)** The survival of mice bearing tumor after different treatments. The data were analyzed using the log-rank (Mantel–Cox) test (*p < 0.05, **p < 0.01, and ***p < 0.001). **(D)** TUNEL-stained assays of tumor tissues after different treatments. Scale bar = 100 μm.

Subsequently, BALB/c nude mice bearing A2780/DDP xenograft were built, and the corresponding treatments were intravenously administered when the tumor size reached about 100 mm^3^. As shown in Figure 7A, the body weight of mice treated with DDP decreased significantly. However, the side effects of DDP on mice were alleviated after DDP was loaded into nanoplatforms, which might be due to the relatively slow release of DDP. The inhibitory effect of TPP-DMON@DDP on tumor growth was significantly greater than that of other treatments (Figure 7B). Further, after relevant treatment, the median survivals were 32, 36, 47, and 57 days in PBS-, DDP-, DMON@DDP-, and TPP-DMON@DDP-treated mice, respectively (Figure 7C). A terminal deoxynucleotidyl transferase dUTP nick end labeling (TUNEL) assay was administered to roughly analyze the apoptosis by detecting DNA fragments (Lv et al., 2020). As shown in Figure 7D, TPP-DMON@DDP-treated tumors were observed with much more fluorescence regions than others, indicating an excellent therapeutic effect in accordance with the result of tumor growth. These results are in agreement with the results of *in vitro* studies. These data demonstrated the potential of TPP-DMON@DDP as a promising agent for DDP-resistant cancer cells.

In addition, DDP-resistant mechanisms in cancer cells also include decreasing drug uptake and increasing drug efflux. DDP loaded and transported by the nanoplatforms can bypass CTR1 through endocytosis and increase the uptake of DDP (Davis et al., 2008; Yao et al., 2020). Furthermore, mitochondria-derived ATP is the energy source for the efflux of anti-tumor drugs mediated by ATP binding cassette (ABC) transporters (Giddings et al., 2021). The treatments that attenuate mitochondrial respiration and inhibit the activity of ABC transporter can promote the accumulation of DDP in cancer cells and increase the response of cancer cells to chemotherapy.

## Conclusion

In summary, we synthesized GSH-responsive nanoplatforms, DMON, modified with TPP, for targeting the mitochondria, resulting in depleting mitochondria GSH and delivery of DDP to the mitochondria. Depletion of mitochondrial GSH by the S-S of DMON disrupted the redox homeostasis of the mitochondria, resulting in the mitochondria of resistant cells being more vulnerable to ROS or DDP. Subsequently, both DDP and DDP-induced ROS attacked mitochondria and caused MMP depolarization, which effectively triggered the mitochondria-mediated apoptosis of DDP-resistant cells. Our findings demonstrate that redox dyshomeostasis of mitochondria induced by the GSH-degradable nanoplatforms exhibits excellent enhancement for DDP cytotoxicity, which provides a promising strategy for exploiting nanoplatforms utility in overcoming chemotherapy resistance.

## Data Availability

The original contributions presented in the study are included in the article/Supplementary Material, Further inquiries can be directed to the corresponding authors.
