# Supplementary material for: Mitochondria-Targeted Mesoporous Organic Silica Nanoplatforms for Overcoming Cisplatin Resistance by Disturbing Mitochondrial Redox Homeostasis
Source: Front Chem. 2022 May 9;10:875818. doi: 10.3389/fchem.2022.875818 (PMC9124779; doi:10.3389/fchem.2022.875818)
Supplement: Supplementary file 1 [file DataSheet1.PDF]

Supplementary Material

Mitochondria-targeted mesoporous organic silica nanoplatforms for overcoming cisplatin resistance by disturbing mitochondrial redox homeostasis

Bin Lv[1,2†], Jingru Ma[3†], Yumeng Wang[1,2], Xinyu Qu[1,2], Junjun Qiu[1,2*], Keqin Hua[1,2*]

[1] Department of Gynecology, Obstetrics and Gynecology Hospital, Fudan University, Shanghai, China

[2] Shanghai Key Laboratory of Female Reproductive Endocrine Related Diseases, Shanghai, China

[3] ENT institute and Department of Otorhinolaryngology, Eye & ENT Hospital, Fudan University, Shanghai, China.

*** Correspondence:**
Junjun Qiu
qiujunjun1113@163.com

Keqin Hua
huakeqin@fudan.edu.cn

1 Supplementary Figures

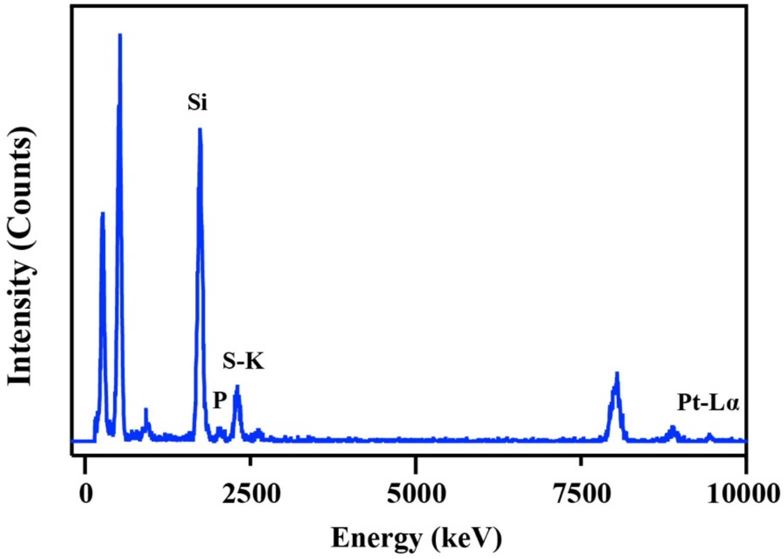

Supplementary Figure 1. The energy dispersive X-ray (EDX) spectrum of TPP-DMON@DDP.

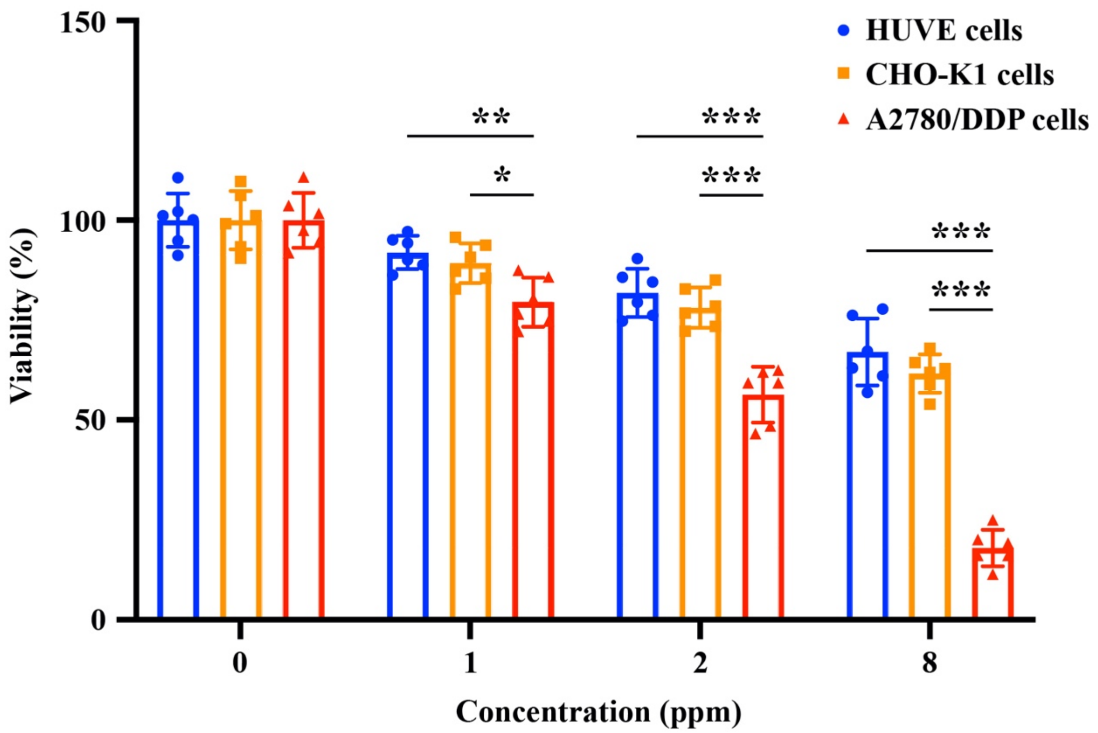

Supplementary Figure 2. Cell viability of HUVE cells and CHO-K1 cells after incubation with TPP-DMON@DDP.

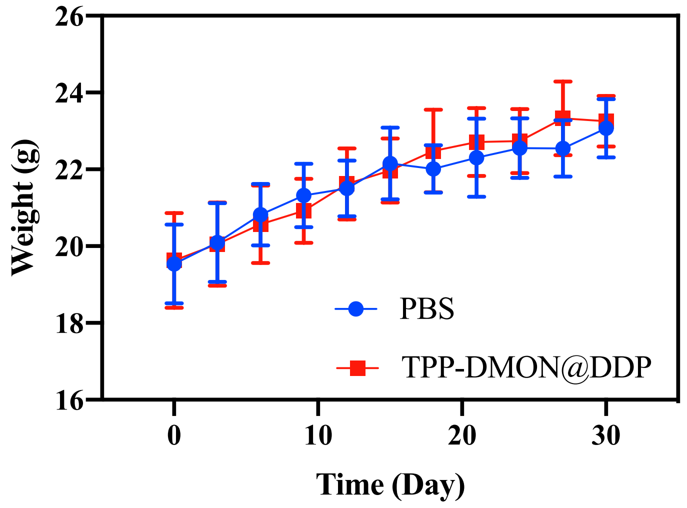

Supplementary Figure 3. Body weight of BALB/c mice injected intravenously with PBS or TPP-DMON@DDP (DDP, 4 mg kg^{-1})

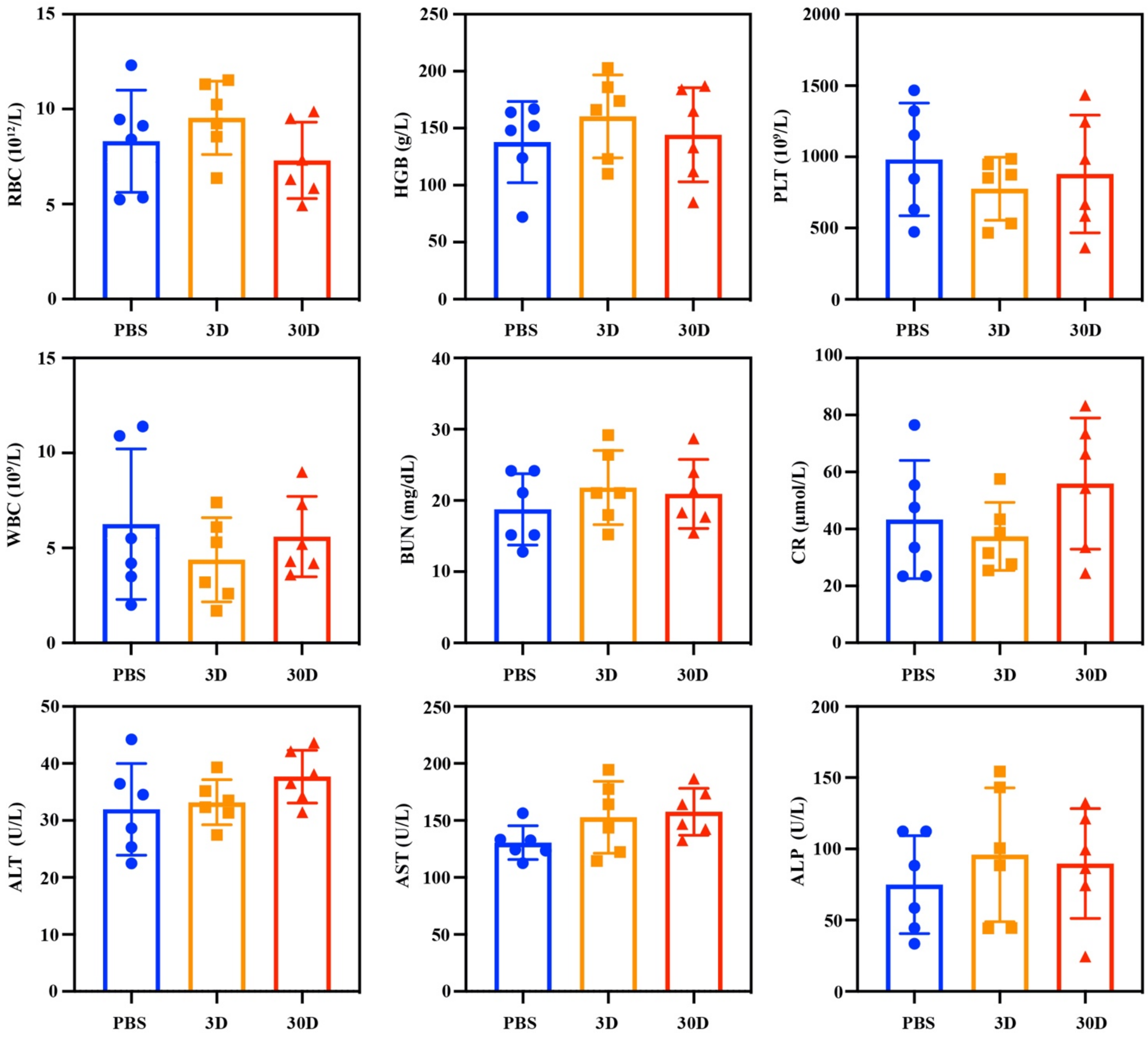

Supplementary Figure 4. Hematological parameters of BALB/c mice after intravenous injection with PBS or TPP-DMON@DDP (DDP, 4 mg kg^{-1}).
